# Supplementary figures and images for: Effects of miR-103 by negatively regulating SATB2 on proliferation and osteogenic differentiation of human bone marrow mesenchymal stem cells
Source: PLoS One. 2020 May 7;15(5):e0232695. doi: 10.1371/journal.pone.0232695 (PMC7205233; doi:10.1371/journal.pone.0232695)

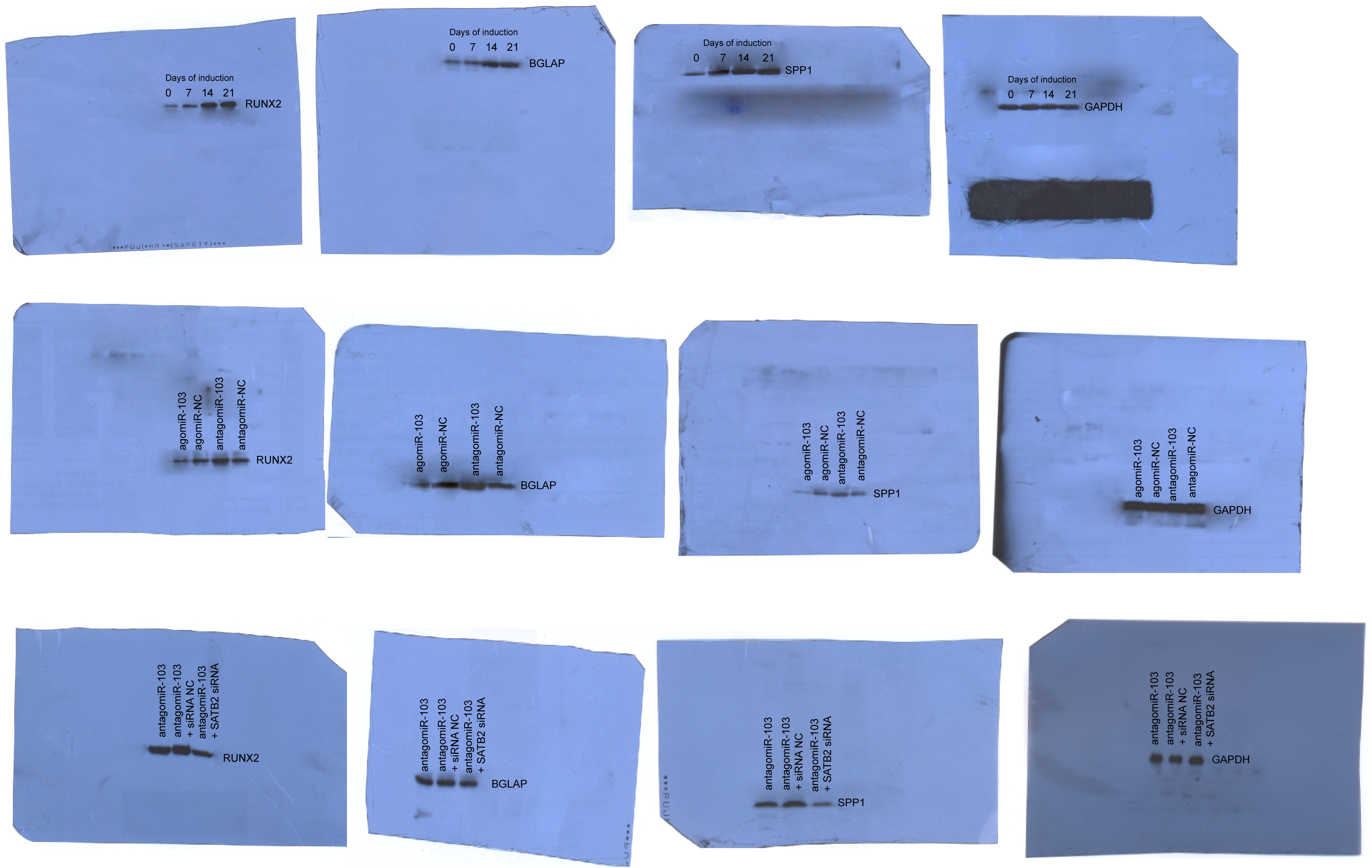

Supplement: S1 Fig — (TIF) [file pone.0232695.s001.tif]
